# Correction: Profiling subcellular localization of nuclear-encoded mitochondrial gene products in zebrafish

**DOI:** 10.26508/lsa.202402628

**Published:** 2024-02-12

**Authors:** Barbara Uszczynska-Ratajczak, Sreedevi Sugunan, Monika Kwiatkowska, Maciej Migdal, Silvia Carbonell-Sala, Anna Sokol, Cecilia L Winata, Agnieszka Chacinska

**Affiliations:** 1 Institute of Bioorganic Chemistry, Polish Academy of Sciences , Poznan, Poland; 2 Centre of New Technologies, University of Warsaw, Warsaw, Poland; 3 ReMedy International Research Agenda Unit, University of Warsaw, Warsaw, Poland; 4 International Institute of Molecular and Cell Biology, Warsaw, Poland; 5 Centre for Genomic Regulation, The Barcelona Institute of Science and Technology, Barcelona, Spain; 6 Department of Developmental Genetics, Max Planck Institute for Heart and Lung Research, Bad Nauheim, Germany; 7 Biomolecular Mass Spectrometry, Max Planck Institute for Heart and Lung Research, Bad Nauheim, Germany; 8 ReMedy International Research Agenda Unit, IMol Polish Academy of Sciences, Warsaw, Poland

## Abstract

The vast majority (99%) of mitochondrial proteins are encoded by the nuclear genome, synthesized in the cytosol and imported into the organelle. Here we study the principles of mitochondrial import in vivo from the RNA perspective using zebrafish.

Article: Uszczynska-Ratajczak B, Sugunan S, Kwiatkowska M, Migdal M, Carbonell-Sala S, Sokol A, Winata CL, Chacinska A (2022 Oct 25) Profiling subcellular localization of nuclear-encoded mitochondrial gene products in zebrafish. Life Sci Alliance 6(1): e202201514. doi: 10.26508/lsa.202201514. PMID: 36283702.

The authors would like to correct an error in the sequences of several primers listed in the Table S2. The corrected sequences have been updated in the table as follows:

mrpl52_F

CGTGGTGGATCAGAGTACGG

mrpl52_R

ACGGCTCTTCTAGCAAACTCC

mrps33_F

CGGGATTCCAGAAAAGGGTGA

mrps33_R

GATTCGAGCACTCAGACGGG

mrpl32_F

TTGCATCGTCTGGAACTCCG

mrpl32_R

TACTGCCAGAGCTGGACCTAA

timm9_F

GTAACGATGGCAGCTCAGGTC

timm9_R

CATGTGGTCTCCTCTGGCTTGA

ndufa8_F

GCTGTCAACTTCTTCAGGCAG

ndufa8_R

CGCAGCTCACCCATGTTAGA

coa6_F

CGTACCTGATAGAGTGAGGAGAG

coa6_R

ACTCTCGCTGGTGTTTCTCG

mcur1_F

AGGGTGTTCAGAGAGTTGAGC

mcur1_R

GGACCATTGCATGAGTGTCG

Table S2 List of primers used in this study.

The authors apologize for any confusion this may have caused.

## Supplementary Material

Reviewer comments

