# Supplementary material for: Correction: Profiling subcellular localization of nuclear-encoded mitochondrial gene products in zebrafish
Source: Life Sci Alliance. 2024 Feb 12;7(5):e202402628. doi: 10.26508/lsa.202402628 (PMC10862497; doi:10.26508/lsa.202402628)
Supplement: Reviewer comments [file LSA-2024-02628_review_history.pdf]

Correction: Profiling subcellular localization of nuclear-encoded mitochondrial gene products in zeb

Barbara Uszczynska Ratajczak, Sreedevi Sugunan, Monika Kwiatkowska, Maciej Migdal, Silvia Carbonell-Sala, Anna Sokol, Cecilia Winata, and Agnieszka Chacinska
DOI: https://doi.org/10.26508/lsa.202402628

Corresponding author(s): Cecilia Winata, International Institute of Molecular and Cell Biology in Warsaw, Poland and Agnieszka Chacinska, The International Institute of Molecular Mechanisms and Machines Polish Academy of Sciences

**Review Timeline:**	Submission Date:	2024-01-29
	Accepted:	2024-02-06

Scientific Editor: Eric Sawey, PhD

Transaction Report:

(Note: With the exception of the correction of typographical or spelling errors that could be a source of ambiguity, letters and reports are not edited. The original formatting of letters and referee reports may not be reflected in this compilation.)

February 6, 2024

RE: Life Science Alliance Manuscript #LSA-2024-02628

Dr. Cecilia Lanny Winata
International Institute of Molecular and Cell Biology in Warsaw, Poland
4 Ks. Trojdena
Warsaw, Mazowieckie 02-109
Poland

Dear Dr. Winata,

Thank you for submitting your Correction entitled "Correction: Profiling subcellular localization of nuclear-encoded mitochondrial gene products in zeb".

Your manuscript will now progress through copyediting and proofing.

IMPORTANT: If you will be unreachable at any time, please provide us with the email address of an alternate author. Failure to respond to routine queries may lead to unavoidable delays in publication.

You can contact the journal office with any questions, contact@life-science-alliance.org

Sincerely,

Eric Sawey, PhD
Executive Editor
Life Science Alliance
http://www.lsajournal.org

February 6, 2024

RE: Life Science Alliance Manuscript #LSA-2024-02628

Dr. Cecilia Lanny Winata
International Institute of Molecular and Cell Biology in Warsaw, Poland
4 Ks. Trojdena
Warsaw, Mazowieckie 02-109
Poland

Dear Dr. Winata,

Thank you for submitting your Correction entitled "Correction: Profiling subcellular localization of nuclear-encoded mitochondrial gene products in zeb".

Your manuscript will now progress through copyediting and proofing.

IMPORTANT: If you will be unreachable at any time, please provide us with the email address of an alternate author. Failure to respond to routine queries may lead to unavoidable delays in publication.

You can contact the journal office with any questions, contact@life-science-alliance.org

Sincerely,

Eric Sawey, PhD
Executive Editor
Life Science Alliance
http://www.lsajournal.org
